# Upregulating sirtuin 6 ameliorates glycolysis, EMT and distant metastasis of pancreatic adenocarcinoma with krüppel-like factor 10 deficiency

**DOI:** 10.1038/s12276-021-00687-8

**Published:** 2021-10-26

**Authors:** Yi-Chih Tsai, Su-Liang Chen, Shu-Ling Peng, Ya-Li Tsai, Zuong-Ming Chang, Vincent Hung-Shu Chang, Hui-Ju Ch’ang

**Affiliations:** 1 National Institute of Cancer Research, National Health Research Institutes, Zhunan, Taiwan; 2 Department of Pathology, National Cheng Kung University Hospital, Tainan, Taiwan; 3 Program for Translation Biology, College of Medical Science and Technology, Taipei Medical University, Taipei, Taiwan; 4 Program for Cancer Biology and Drug Discovery, College of Medical Science and Technology, Taipei Medical University, Taipei, Taiwan; 5 Department of Oncology, School of Medicine, College of Medicine, National Cheng Kung University, Tainan, Taiwan

**Keywords:** Pancreatic cancer, Epithelial-mesenchymal transition, Cancer metabolism, Translational research

## Abstract

Krüppel-like factor 10 (KLF10) is a tumor suppressor in multiple cancers. In a murine model of spontaneous pancreatic adenocarcinoma (PDAC), additional KLF10 depletion accelerated distant metastasis. However, Klf10 knockout mice, which suffer from metabolic disorders, do not develop malignancy. The mechanisms of KLF10 in PDAC progression deserve further exploration. KLF10-depleted and KLF10-overexpressing PDAC cells were established to measure epithelial-mesenchymal transition (EMT), glycolysis, and migration ability. A murine model was established to evaluate the benefit of genetic or pharmacological manipulation in KLF10-depleted PDAC cells (PDACshKLF10). Correlations of KLF10 deficiency with rapid metastasis, elevated EMT, and glycolysis were demonstrated in resected PDAC tissues, in vitro assays, and murine models. We identified sirtuin 6 (SIRT6) as an essential mediator of KLF10 that modulates EMT and glucose homeostasis. Overexpressing SIRT6 reversed the migratory and glycolytic phenotypes of PDACshKLF10 cells. Linoleic acid, a polyunsaturated essential fatty acid, upregulated SIRT6 and prolonged the survival of mice injected with PDACshKLF10. Modulating HIF1α and NFκB revealed that EMT and glycolysis in PDAC cells were coordinately regulated upstream by KLF10/SIRT6 signaling. Our study demonstrated a novel KLF10/SIRT6 pathway that modulated EMT and glycolysis coordinately via NFκB and HIF1α. Activation of KLF10/SIRT6 signaling ameliorated the distant progression of PDAC.

**Clinical Trial Registration:** ClinicalTrials.gov. identifier: NCT01666184.

## Pancreatic cancer: Possible treatment based on tumor suppressor genes

Boosting the activity of a tumor suppressor pathway in pancreatic cancer may reduce the risk of metastasis. Reduced expression of a tumor suppressor gene called Krüppel-like factor 10 (KLF10) is found in breast, lung and pancreatic cancers. KLF10 deficiency in pancreatic cancer is linked to accelerated cancer progression and metastasis, but the underlying mechanisms are unclear. In experiments on mouse models and human cell cultures, Hui-Ju Ch’ang at the National Institute of Cancer Research in Zhunan, Taiwan, and co-workers found that KLF10 binds to and upregulates another tumor suppressor gene, SIRT6. This second gene mediates KLF10 activity, including its role in regulating glucose metabolism and a process that transforms epithelial cells into cells with invasive, migratory characteristics. Overexpressing SIRT6 in KLF10-deficient cells reversed glycolysis, migratory cell behavior and reduced metastasis, suggesting a potential treatment option.

## Introduction

Deregulation of transforming growth factor (TGF)-β signaling is a common feature of pancreatic adenocarcinoma (PDAC)^1^. TGFβ/Smad signaling suppresses malignant growth early in epithelial tumorigenesis but promotes metastasis in later stages. This complexity and intratumor genetic heterogeneity make the resulting effects of TGFβ inhibition on cancer cell compartments difficult to predict^2^.

Krüppel-like factor 10 (KLF10) is an early response gene of TGFβ signaling that provides positive feedback toward the antiproliferative effect of TGFβ^3,4^. Recent studies, including ours, have revealed the downregulation of KLF10 in several advanced cancers, including breast, lung, and pancreas cancers, which results in the development of cancer metastasis^5–7^. KLF10 limits epithelial-mesenchymal transition (EMT) by suppressing Slug transcriptionally and activating stromal cell-derived factor-1/CXCR4 signals in lung and pancreatic cancers^6,7^. Distinguishing the antiproliferative and prometastatic functions of TGFβ makes KLF10 a potential target for the development of therapies for cancers with aberrant TGFβ pathways, such as PDAC.

Additional KLF10 depletion in Kras-mutated (KC) mice revealed the accelerated progression of PDAC^6^, whereas KLF10 knockout mice did not develop any pancreatic malignancies^3^. Studies of the livers of KLF10-deficient mice have indicated that KLF10 has a significant role in regulating genes that are involved in glucose metabolism^8,9^. Our preliminary study used the chromatin immunoprecipitation (ChIP)-chip assay and found that 550 genes from eight networks were physically associated with the KLF10 protein^10^. Sirtuin 6 (SIRT6) is one of a few downstream genes that are bound by KLF10 and govern cancer metabolism and tumor progression^11^. In this study, we observed that KLF10 bound to the promoter and transcriptionally regulated SIRT6. The loss of KLF10 led to reduced expression of SIRT6 and contributed to elevated glycolysis, the EMT phenotype, and distant metastasis of PDAC. Glycolysis activity and the EMT phenotype of PDAC cells with KLF10 mRNA silencing were reversed by genetic SIRT6 overexpression. Linoleic acid (LA), which is a long-chain fatty acid, increased SIRT6 activity but not that of other sirtuins^12^ and reversed aberrant glucose metabolism, EMT, and distant metastasis induced by KLF10 deficiency in PDAC.

## Materials and methods

### Cell culture and chemicals

The human pancreatic cancer cell lines Panc-1 (BCRC Cat# 60284, RRID:CVCL_0480), ASPC-1 (BCRC Cat# 60494, RRID:CVCL_0152), and MiaPaCa (BCRC Cat# 60139, RRID:CVCL_0428) were purchased from the Bioresource Collection and Research Center, Taiwan. All the cell lines were authenticated by DNA fingerprinting every three years. Panc-1, ASPC-1 and MiaPaCa cells were labeled with firefly luciferase plasmid vectors (Panc-1-Luc, ASPC-1-Luc, and MiaPaCa-Luc), which were provided by Dr. Kelvin K.C. Tsai of Taipei Medical University, Taiwan. Linoleic acid (LA) was purchased from Sigma-Aldrich (Cat: 366633-5G; St. Louis, MO, USA). In addition, 1,4-dihydrophenonthrolin-4-one-3-carboxylic acid (DPCA) and phorbol ester (PMA) were purchased from Cayman (No. 712220; Ann Arbor, MI, USA) and Merck (P1585; Kenilworth, NJ, USA), respectively.

### Cell migration, invasion, and trajectory analysis

To assay cell migration and invasion, 5 × 10^4^ cancer cells were suspended in 10% fetal bovine serum (FBS) that contained culture medium and were seeded into the top chambers of a Transwell insert (Corning Costar, Corning, NY, USA) with or without Matrigel coating (Corning Costar). The cells were allowed to migrate for 16 h toward the bottom chambers that contained the culture medium plus 10% FBS. The filters of the Transwell insert were then fixed and stained with Giemsa. Migratory or invasive cells on the filters were imaged and quantified by using ImageJ (RRID:SCR_003070) (National Institute of Health, USA). For manual cell tracking, time-lapse photometry was performed to assay cell migration. Each sequential image was examined after seeding at 15-min intervals from 48 to 52 h using the cell-tracking application in ImageJ Pro Plus 5.0, as previously described^13^. Migration distance was measured using the *XY* coordinates from the origin. Plots of cell trajectories that emanated from the origin and the net displacement from the origin were analyzed as previously reported^14^.

### Glucose uptake, lactate production, and mitochondrial metabolism by Seahorse

Glucose uptake was measured using the Glucose Uptake Assay Kit (BioVision, Milpitas, CA, USA) according to the manufacturer’s instructions. Cell lysates were collected and assayed for lactate levels using a lactate assay kit (K627-100; BioVision) according to the manufacturer’s instructions. The optical density values of the cell lysates were analyzed for 24 h. Lactate production was calculated from the standard curve and normalized to the cell lysate concentration. The cells were seeded at an optimized density and incubated for 24 h. Metabolic profiles were generated using the Seahorse XF96 extracellular Flux analyzer (Seahorse Bioscience, Santa Clara, CA, USA) as previously described^15^. The baseline oxygen consumption rate and extracellular rate of acidification were determined after replacing the growth medium with assay medium containing the inhibitors or vehicle, according to the manufacturer’s protocol^16^.

### Animals and the murine liver metastasis model

Mice were housed at the animal core facility of the National Health Research Institutes (NHRI), Taiwan. The facility was approved by the National Association for Accreditation of Laboratory Animal Care, Taiwan, and is maintained in accordance with the regulations and standards of the NHRI Animal Council’s procedural and ethical guidelines. Nonobese diabetic (NOD)/severe combined immunodeficient (SCID) mice (NOD. CB17-*Prkdcscid*/NcrCrlBltw; IMSR Cat# ARC:NODSCID, RRID:IMSR_ARC:NODSCID) were purchased from the National Laboratory Animal Center. For the murine model of liver metastasis, the mice were anesthetized and fixed in the supine position. A small left abdominal flank incision was made, and the spleen was exteriorized. A 1-mL syringe with a 29-gauge needle in which PDAC cells were suspended in phosphate-buffered saline at a concentration of 1 × 10^6^ cells/80 μL was inserted into the spleen. To prevent tumor cell leakage and bleeding, a cotton swab was held over the injection site for 1 min before the spleen was excised. The wound was sutured with 5–0 black silk (Chromic, CC125). For LA treatment, 0% to 1.5% LA dissolved in 0.1% EtOH was added to the daily water of the mice from 2 weeks before tumor injection for 6–8 weeks until sacrifice. Every experiment was repeated independently at least twice. KC mouse specimens for immunohistochemistry of the normal pancreas and PDAC tumors were provided by Dr. Tze-Sing Huang of the National Institute of Cancer Research, NHRI, Taiwan.

### Immunoblot analysis

Cell extracts were prepared in lysis buffer (RIPA Cell Lysis Buffer 5×, RP05–100) that contained a 1 × protease inhibitor mixture (Complete, Roche, Basel, Switzerland) and 1 mM phenylmethylsulfonyl fluoride. For western blot analysis, cell extracts were subjected to sodium dodecyl sulfate-polyacrylamide gel electrophoresis. The electrotransferred membrane (Amersham, Darmstadt, Germany; Hybond-PVDF NO 1060023) was then incubated with the secondary antibody (IRDye 680RD Donkey anti-Mouse IgG, RRID AB_2716622; IRDye 800CW Donkey anti-Rabbit IgG, RRID AB_2715510) and developed with a LI-COR Odyssey system (LI-COR, Bad Homburg, Germany). Integrated optical density quantification was performed using Image Studio v 5.2. We used E-cadherin (1:1000, Cat# ab76319, RRID:AB_2076796; Abcam Cambridge, UK), Slug (1:500, Cat# 9585, RRID:AB_2239535; Cell Signaling Technology, Danvers, MA;), vimentin (1:1000, Abcam Cat# ab92547, RRID:AB_10562134), pyruvate dehydrogenase kinase isozyme 1 (PDK1; 1:1000, Abcam Cat# 1624-1 ab202468, RRID:AB_562190), pyruvate kinase isozyme M2 (PKM2; 1:2000, Cell Signaling Technology Cat# 4053, RRID:AB_1904096), and glucose transporter 1 (Glut1; 1:1000, Abcam Cat# ab32551, RRID:AB_732605) to detect EMT and glycolysis protein markers. The KLF10 antibody was purchased from Abcam (1:1000, Abcam Cat# ab73537, RRID:AB_1640621). A β-actin antibody (Sigma-Aldrich Cat# A5441, RRID:AB_476744) at a 1:5000 dilution was used as the control. SIRT6 (1:1000, Cell Signaling Technology Cat# 12486, RRID:AB_2636969), HIF1α (1:1000, Cat# GTX127309, RRID:AB_2616089; Gene Tex, Irvine, CA, USA), and NFκB (1:1000, Abcam Cat# ab109440, RRID:AB_10866623) antibodies were used to study the downstream signaling of KLF10.

### Immunohistochemical staining

Paraffin-embedded tissue sections were deparaffinized in xylene, rehydrated through a series of ethanol dilutions, and boiled for 15 min in 10 mM citrate buffer at pH 6.0. Endogenous peroxidase activity was suppressed using a peroxidase block (Novolink, Chino, CA, USA, RE7148) for 5 min. The tissue sections were then blocked using protein blocks (Novolink, RE-7159) and incubated overnight at 4 °C with antibodies against KLF10 (1:400, mouse monoclonal antibody, LTK BioLaboratories, Taoyuan, Taiwan), SIRT6 (1:200, Abcam Cat# ab62738, RRID:AB_956299), PDK1 (1:400, Abcam Cat# 1624-1 ab202468, RRID:AB_562190), and PKM2 (1:400, Cell Signaling Technology Cat# 4053, RRID:AB_1904096). Antibody detection was performed using the Novolink Max Polymer Detection System (RE7280-K, Leica Biosystems Newcastle Ltd, Newcastle upon Tyne, UK).

### Transfection and transduction

The construction of KLF10 expression vectors has been described previously^17^. The stable downregulation of scramble and KLF10 plasmids (TRCN0000318921) in Panc-1 or ASPC-1 cells was achieved using a retrovirus-mediated RNA interference system (pSUPER.retro.puro, VEC-PRT-0001, OligoEngine, Seattle, WA, USA) and shRNAs purchased from the National RNAi Core Facility of Academic Sinica (Taipei, Taiwan): 5′-GAACCCTCTCAAGTGTCAAAT-3′. Infected cell populations were selected using 2 mg/mL puromycin. The efficacy was confirmed through western blotting. We obtained at least five stable clones of Panc-1 or ASPC-1 cells by using the most efficient KLF10-targeting shRNA constructs. Clones with optimal KLF10 protein reduction and migratory ability were chosen to perform the following experiments (Supplementary Fig. 1a). The KLF10 cDNA cloned in the pcDNA3.1+ plasmid vector (RRID: Addgene_117272) was retrieved through BamHI and EcoRI restriction enzyme digestion and subcloned into BamHI and EcoRI enzyme-digested pLVX-TetOne vector plasmids (Takara Bio USA, Inc., Mountain View, CA, USA). The resulting pLVX-KLF10 plasmids with appropriate inserts were verified by using polymerase chain reaction (PCR), restriction enzyme digestion, and signature enzyme digestion. MiaPaCa was transiently transfected with pLVX-KLF10 plasmids or empty vectors and tested for transgene induction with doxycycline. The cloned transgene was cotransfected with lentiviral supernatants that were produced from Lent-X Packaging Single Shots into Lenti-X 293T cells (632180, Takaro Bio USA, Inc). MiaPaCa cells were transduced with TetOne virus and selected using puromycin (0.2 μg/mL, Sigma) for 2 weeks. The surviving fraction of cells was then expanded and screened for KLF10 expression after doxycycline (500 ng/mL) treatment for 24 h. The KLF10-expressing cells were then seeded in 96-well plates and subjected to single-cell cloning. Each clonal population of cells was subsequently screened for KLF10 protein expression through western blotting. Forced expression of KLF10 in PDACshKLF10 cells was achieved by transient transduction of pcDNA3.1-HA-KLF10^18^ by Lipofectamine 3000 (Thermo Fisher Scientific, MA, USA) for 48 h. To determine whether SIRT6 is a major downstream mediator of KLF10, a stable KLF10 mRNA silencing clone was transiently transduced with SIRT6-Flag plasmids (pcDNA3.1-FLAG-SIRT6, Addgene #13817, Watertown, MA, USA) using Lipofectamine 3000 (L3000015, Thermo Fisher Scientific) for 48 h.

### Chip-PCR and reverse transcription PCR analysis

The EZ ChIP chromatin immunoprecipitation kit (#17–371 Sigma-Aldrich, St. Louis, MO, USA) was used according to the manufacturer’s protocol. Briefly, the cells were transfected with scrambled plasmid or SIRT6 plasmid for 48 h and treated with 1% formaldehyde to cross-link proteins to DNA. The cells were lysed with protease inhibitors, sonicated to shear DNA into fragments and incubated with antibodies against KLF10 or anti-rabbit IgG overnight. The purified DNA and input genomic DNA were analyzed using real-time PCR (RT-PCR). The primer sequences for Sirt6 were as follows: forward: 5′-TGTGTTGTCCAGAGGTGAGG, reverse: 5′-TGCAAGCCCTCTACTGATCCC. RNA was extracted using the RNeasy plus kit (QIAGEN, Hilden, Germany) after transfection. For RT-PCR, an ImPromII reverse transcription kit (A3800 Promega, Madison, WI, USA) and GoTaq Mix (M 7122, Promega) were used as recommended by the manufacturer. The primers used were SIRT6 left: TGTGTTGTCCAGAGGTGAGG, SIRT 6 right: TGCAAGCCCTCTACTGATCCC; glyceraldehyde 3-phosphate dehydrogenase (GAPDH) left: ACCCACTCCT CCACCTTTGA, GAPDH right: TGTTGCTGTAGCCAAATTCGTT.

### Plasmid construction and promoter luciferase assay

Proximal promoter fragments of SIRT6 that spanned −1807 to +76 and contained six specific protein 1 binding sites were cloned upstream of the luciferase gene in the pGL3-based luciferase expression plasmid (Promega #E175A; Fig. 3a). A mutated fragment of the SIRT6 promoter (−80 ~ −50 deletion) was generated. Before cloning, each fragment was amplified using PCR with specific primers, and restriction enzyme cutting sites were designed. Panc-1 cells were transfected with individual SIRT6 reporter constructs and cotransfected with pRLTK (E2241, Promega), which constitutively expresses Renilla luciferase, to normalize transfection efficiency. The promoter activities with or without KLF10 treatment were determined using a dual-luciferase assay kit (E1910, Promega). Data are presented as the ratio of promoter reporter luciferase activity to control vector pGL3-Enhancer luciferase activity.

### In vivo imaging system

Luciferin, the substrate of luciferase, was injected intraperitoneally into mice at a dose of 125 mg/kg of body weight. The mice were then anesthetized and placed on the imaging stage of the in vivo imaging system (IVIS) apparatus in the supine position. Images were collected every few minutes from 10 to 30 min after luciferin injection using IVIS (Xenogen, Hopkinton, MA, USA), and the photons emitted from the tumor and its surroundings were quantified using Living Image Software (Xenogen). To minimize the limitations of luminescence imaging, the mice were all placed in the supine position during measurement. Tumor necrosis with related low signal intensity was excluded through histologic examination.

### Patient specimens and statistics

Pancreatic tumor specimens were obtained from 110 of 147 patients with curatively resected pancreatic cancer enrolled in a randomized phase III clinical trial. (ClinicalTrials.gov. identifier: NCT 00994721). Eligible patients were 20–75 years old, with adequate organ function and serum CA19–9 levels less than 2.5× the institutional upper limit. Only 105 specimens were optimal for evaluating KLF10 immunostaining (ClinicalTrials.gov. identifier: NCT01666184). The tissue slides were examined independently by two observers (S.L.P. and C.C.) who were masked to both the clinical and pathological data. Immunopositivity was assessed regarding cellular localization, intensity, and distribution. The expression of biomarkers was quantified using a visual grading system based on the extent of staining (percentage of positive tumor cells graded on a scale of 0–3: 0, 0%; 1, 1%–30%; 2, 31%–60%; and 3, >60%) and the intensity of staining (graded on a scale of 0–3: 0, no staining; 1, weak staining; 2: moderate staining; and 3, strong staining). The combination of the extent (E) and intensity (I) of staining was obtained by calculating E × I to determine the extent-intensity (EI), which varied from 0 to 9. The mean EI score was calculated for each pancreatic cancer specimen. For the statistical analysis of KLF10, EI scores of 0–1 were considered low expression, and EI scores > 1 were considered high expression. The correlations of EI scores between KLF10 and SIRT6, PKM2 and PDK1 were analyzed in 29, 10, and 10 patients, respectively, from the 110-member cohort mentioned above.

SPSS (v 22.0; RRID:SCR_002865) was used for statistical analyses of group comparisons of normally distributed data by the independent Student’s *t*-test or one-way analysis of variance. Pearson’s correlation analysis was used to determine the correlation between the expression of two molecules. Kaplan–Meier analysis and the log-rank test were used to analyze overall survival and distant metastasis-free survival. Statistical differences were considered significant at *p* < 0.05, *p* < 0.01, and *p* < 0.005.

## Results

### Loss of KLF10 was correlated with enhanced migratory ability and distant metastasis of PDAC

**Fig. 1:**
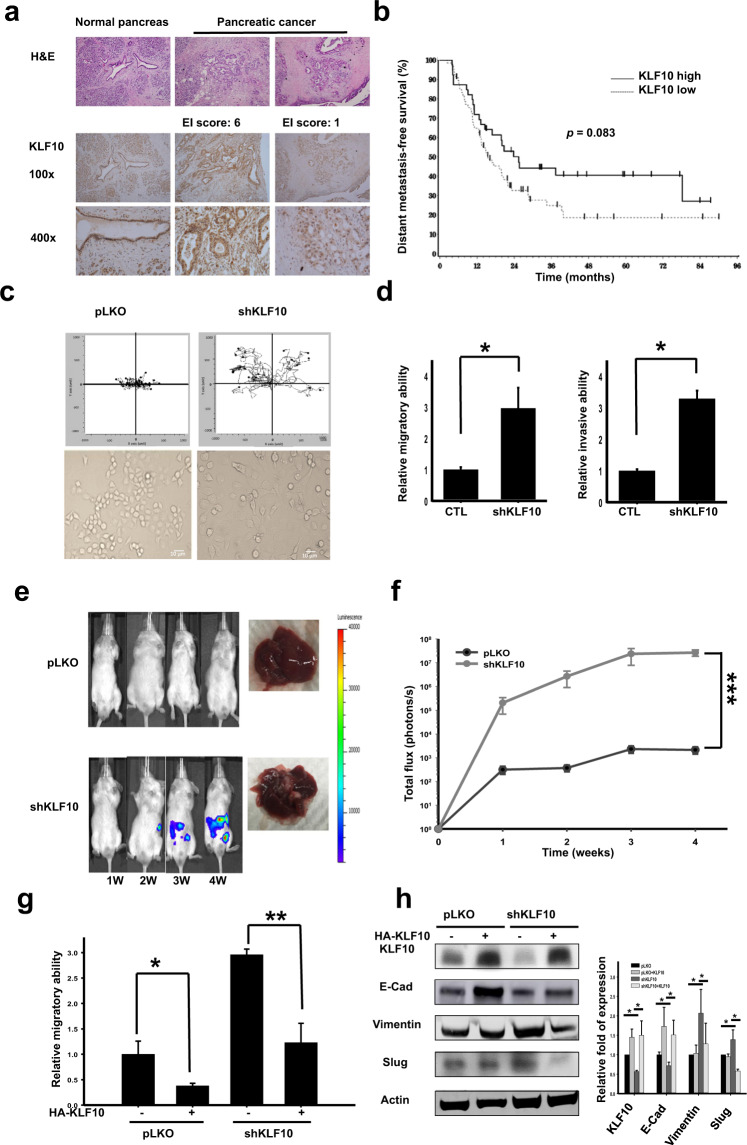
Loss of Krüppel-like factor 10 (KLF10) correlated with enhanced migratory ability, epithelial-mesenchymal transition phenotypes, and distant metastasis of pancreatic adenocarcinoma (PDAC). **a** Representative H&E (upper panel) and immunohistochemistry of KLF10 (middle and lower panels) in resected normal pancreas (left panel) and PDAC tissues of high (middle panel) and low (right panel) extent-intensity (EI) score immunostaining. The photos in the upper, middle, and lower panels are enlarged by ×100, ×100, and ×400, respectively. **b** Distant metastasis-free survival (DMFS) curves of 105 patients with curatively resected PDAC. The median DMFS was 15.5 and 25.4 months for patients with low and high KLF10 expression, respectively (*p* = 0.083). **c** Cumulated cell trajectory assay of Panc-1pLKO (left upper panel) and Panc-1shKLF10 (right upper panel) cells from 20 cell lines. Representative morphology of Panc-1pLKO (left lower panel) and Panc-1shKLF10 cells (right lower panel) enlarged by ×400 under bright field. **d** Cumulative migration (left panel) and invasion (right panel) assays of Panc-1pLKO (CTL) and Panc-1shKLF10 cells. Data are presented as the mean ± standard error (SE; * signifies *p* < 0.05). The experiments were repeated independently three times. **e** Representative in vivo imaging system (IVIS) images of mice injected with ASPC-1pLKO (upper panel) and ASPC-1shKLF10 (lower panel) for 1–4 weeks. Representative liver specimens with tumor nodules are displayed on the right side of each panel. **f** Cumulative IVIS signals of at least six mice in each experimental group injected with ASPC-1pLKO (black) and ASPC-1shKLF10 (gray) are shown at the time after injection. Data are presented as the mean ± SE (*** signifies *p* < 0.005). **g** Cumulative migratory assay data of Panc-1pLKO and Panc-1shKLF10 cells with and without forced replacement of KLF10 (* and ** represent *p* < 0.05 and *p* < 0.01, respectively). The experiments were repeated three times. **h** Representative immunoblots of the indicated proteins in Panc-1pLKO and Panc-1shKLF10 cells with and without forced replacement of KLF10. Quantitative analysis of at least three independent experiments is shown in addition to immunoblots. β-Actin was used as the internal control.

From the pancreatic tumor specimens of 105 patients who received curative intent surgery, 66 (62.9%) patients presented a low expression of KLF10, according to the definition described in the “Materials and methods” section (Fig. 1a, right panel). Distant metastasis-free survival was shorter in patients with low KLF10 expression (*p* = 0.083, Fig. 1b). To evaluate the role of KLF10 in pancreatic cancer metastasis, we established stable clones of Panc-1 and ASPC-1 cells with KLF10 mRNA silencing (Panc-1shKLF10 [Supplementary Fig. 1a, lower panel], and ASPC-1shKLF10) and MiaPaCa cells with inducible KLF10 overexpression given their inherent expression of KLF10 (Supplementary Fig. 1a, upper panel). A cell trajectory study revealed that both the accumulated and oriented migration distances of Panc-1shKLF10 cells increased compared to those of Panc-1pLKO cells (Fig. 1c, upper panel and Supplementary Fig. 1b, left panel). Elevated migratory ability was detected in both Panc-1shKLF10 and ASPC-1shKLF10 cells (Fig. 1d, left panel; Supplementary Fig. 1b, right panel).

To avoid signal interference of the primary tumor and incidental peritoneal contamination during orthotopic tumor implants, we adopted a liver metastatic model through splenic tumor injection followed by splenectomy, as described previously^19^. The murine model of liver metastasis presented significantly enhanced luminescence signals and metastatic nodules in the liver of mice with KLF10 mRNA silencing versus the control with both ASPC-1 and Panc-1 cells (Fig. 1e, f; Supplementary Fig. 1c, d). Forced expression of KLF10 in Panc-1shKLF10 cells suppressed the migratory ability (Fig. 1g). In parallel, overexpressing KLF10 in MiaPaCa cells suppressed the migratory ability of PDAC cells (Supplementary Fig. 1e).

KLF10 deficiency correlated with increased migration and distant metastasis of pancreatic cancer cells.

### KLF10 regulated epithelial-mesenchymal transition

Cell morphology became elongated after KLF10 mRNA silencing (Fig. 1c, lower panel), with enhanced Transwell invasion ability in Panc-1 cells (Fig. 1d, right panel). Immunoblots revealed that the E-cadherin level was significantly reduced and that the mesenchymal protein expression levels were elevated in response to KLF10 mRNA silencing (Fig. 1h). Similar observations were noted in ASPC-1shKLF10 (Supplementary Fig. 1f). Forced expression of KLF10 in Panc-1shKLF10 cells and overexpression of KLF10 in MiaPaCa elevated E-cadherin levels and downregulated mesenchymal proteins (Fig. 1h and Supplementary Fig. 1g).

Loss of KLF10 is correlated with enhanced EMT phenotypes of PDAC.

### KLF10 loss was correlated with glycolysis in pancreatic cancer cells

**Fig. 2:**
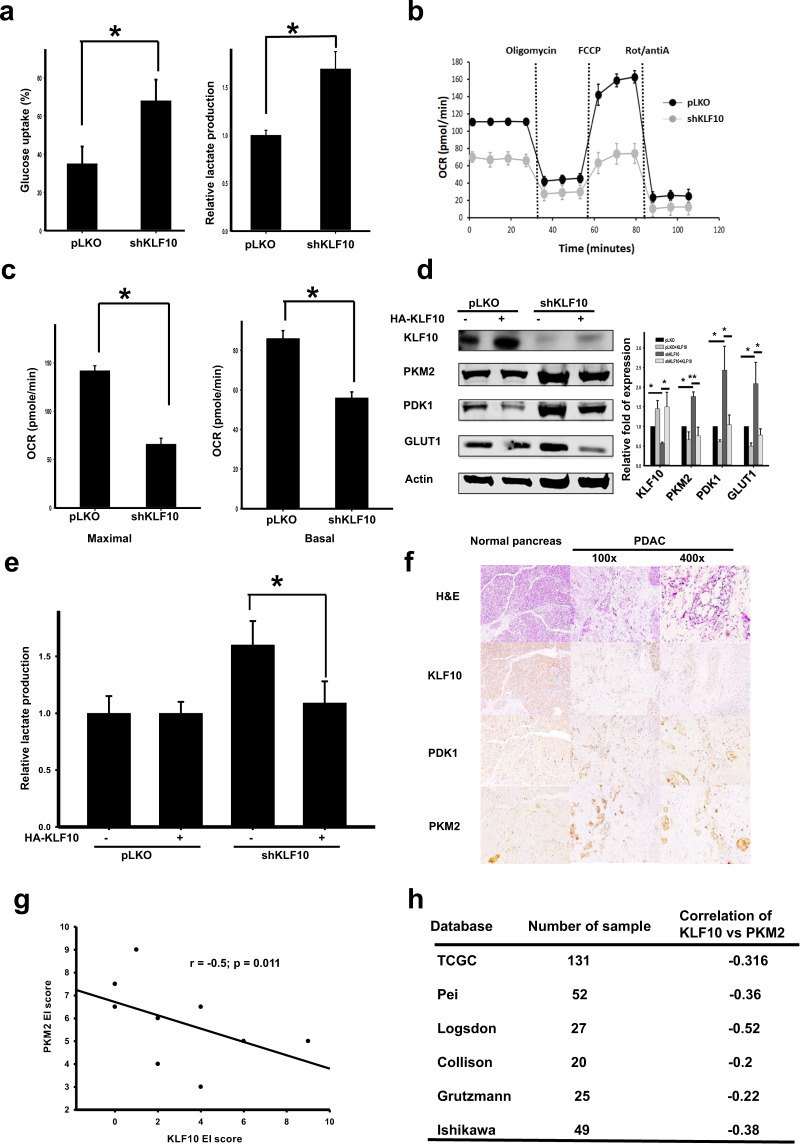
Loss of KLF10 correlated with enhanced glycolysis in PDAC. **a** Cumulated data of glucose uptake (left panel) and lactate production assays (right panel) in Panc-1pLKO and Panc-1shKLF10 cells. Data are presented as the mean ± SE (* signifies *p* < 0.05). The experiments were repeated three times. **b** Representative oxygen consumption rate in Panc-1pLKO (black) and Panc-1shKLF10 (gray) cells in response to oligomycin, carbonyl cyanide-p-trifluoromethoxyphenyl-hydrazon (FCCP), antimycin A (antiA), and rotenone (Rot). The results were normalized according to cell number and are presented as the mean ± SE (*n* = 3). **c** Basal and maximal respiration were determined in the same experimental setting for Panc-1pLKO and Panc-1shKLF10 cells in (**b**). **d** Representative immunoblots of KLF10 and glycolytic enzyme expression in Panc-1pLKO and Panc-1shKLF10 cells with and without KLF10 replacement. β-Actin was used as the internal control. Quantitative analysis of at least three independent experiments is shown beside the immunoblots. **e** Cumulated lactate production assay data of Panc-1pLKO and Panc-1shKLF10 cells with and without KLF10 replacement. Data are presented as the mean ± SE (* signifies *p* < 0.05). **f** Representative hematoxylin and eosin (H&E) staining and immunohistochemistry of KLF10, PKM2, and PDK1 in nontumor normal pancreas and pancreatic tissues (×100 and ×400 for the middle and right panels, respectively) from patients with curatively resected PDAC. **g** Correlation of immunolabeling of KLF10 with PKM2 from the pancreatic tissues of 10 patients from the cohort mentioned in the “Materials and methods” section of PDAC patients who underwent curative resection. The correlation coefficient = −0.5, *p* = 0.011. **h** Representative six databases from Oncomine with correlated levels of KLF10 and PKM2 transcripts.

Given the close association between KLF10 and metabolic disease as reported previously^8,9^, we measured the glycolysis activity of PDAC cells with genetically manipulated KLF10. Glucose uptake, lactate production and glycolytic activity were enhanced over twofold in Panc-1shKLF10 cells (Fig. 2a; Supplementary Fig. 2a, b). Similar observations were noted in ASPC-1shKLF10. Reduced mitochondrial oxidative phosphorylation, basal respiration, and maximal respiration capacity were noted in Panc-1shKLF10 cells compared with Panc-1pLKO cells (Fig. 2b, c). KLF10 deficiency in Panc-1 cells correlated with increased glycolytic enzymes, including PKM2, PDK1, and Glut1 (Fig. 2d). Glycolytic enzymes and lactate production in Panc-1shKLF10 cells were downregulated with forced expression of KLF10 (Fig. 2d, e). In MiaPaCa cells overexpressing KLF10, lactate production and glycolytic enzyme levels were reduced compared with those of the control cells (Supplementary Fig. 2c, d). Pancreatic tumor tissues from KC mice revealed a loss of KLF10 immunolabeling and elevated PKM2 and PDK1 expression (Supplementary Fig. 2e). An immunohistochemical study of KLF10, PKM2, and PDK1 in the PDAC tissue of ten patients showed a negative correlation between KLF10 and glycolytic enzymes (Fig. 2f, g; Supplementary Fig. 2f). Data from Oncomine indicated a moderate inverse correlation of KLF10 with glycolytic enzymes, including PKM2 and PDK1 (Fig. 2h; Supplementary Fig. 2g).

In summary, loss of KLF10 leads to enhanced glycolysis, which parallels the metastatic progression of PDAC.

### KLF10 transcriptionally upregulated SIRT6

**Fig. 3:**
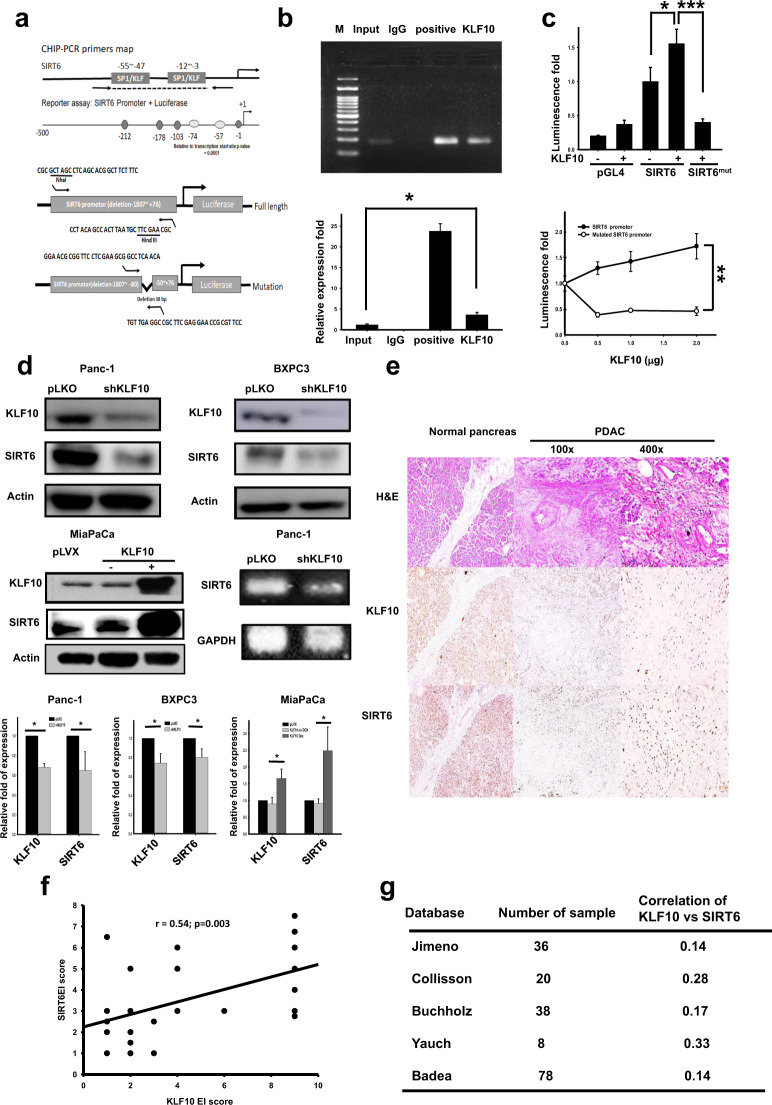
KLF10 bound to the promoter and transcriptionally upregulated sirtuin 6 (SIRT6). **a** Primer maps of the SIRT6 promoter for the chromatin immunoprecipitation-polymerase chain reaction (PCR) assay (upper panel) and luciferase reporter assay (middle and lower panels). **b** Upper panel: DNA fragments of Panc-1 cells immunoprecipitated with KLF10 (KLF10), IgG (IgG), or positive control followed by PCR amplification of the SIRT6 promoter region that contains KLF10 binding sites (input), as described in the “Materials and methods” section. Lower panel: Quantitative PCR of the SIRT6 promoter region using samples from Fig. 3b, upper panel (* denotes *p* < 0.05). **c** Upper panel: SIRT6 promoter luciferase reporter activity in Panc-1 cells with or without KLF10 treatment by using wild-type or mutated SIRT6 promoter plasmids, as described in the “Materials and methods” section. Each bar represents the mean ± SE from at least two experiments (* and *** denote *p* < 0.05 and 0.005, respectively). Lower panel: SIRT6 promoter luciferase reporter activity in Panc-1 cells after various KLF10 treatments using wild-type (black) or mutated (gray) plasmids of the SIRT6 promoter as described in the “Materials and methods”. Each point represents the mean ± SE from at least two experiments (** denotes *p* < 0.01). **d** Representative immunoblots of KLF10 and SIRT6 expression in Panc-1 (left upper panel) and BXPC-3 (right upper panel) cells with and without KLF10 mRNA silencing. MiaPaCa (left lower panel) cells with and without doxycycline (Dox) to induce KLF10 mRNA overexpression. Right lower panel: RNA level of SIRT6 in Panc-1shKLF10. Glyceraldehyde 3-phosphate dehydrogenase (GAPDH) was used as the control. Quantitative analysis is shown below the blots. **e** Representative H&E staining (upper panel) and immunohistochemical staining of KLF10 (middle panel) and SIRT6 (lower panel) in the nontumor normal (left panel, ×100) and pancreatic cancer tissues (middle and right panels, ×100 and ×400, respectively) from patients with PDAC who underwent curative resection. **f** Correlation between the KLF10 expression level and SIRT6 in pancreatic tissue from 29 patients of the cohort mentioned in the “Materials and methods” with PDAC who underwent resection. Correlation coefficient = 0.54; *p* = 0.003. **g** Representative five databases from Oncomine display a positive correlation between KLF10 and SIRT6 transcripts.

By using the ChIP-chip assay^20^, we discovered that SIRT6, which governs metabolism and tumorigenesis, is a candidate gene that is regulated by KLF10. By using ChIP-PCR and qPCR, we noted that KLF10 physically bound to the promoter of SIRT6 (Fig. 3a, upper panel, and 3b). Furthermore, the luciferase reporter assay showed that KLF10 positively regulated SIRT6 transcriptionally (Fig. 3a, middle and lower panels, and 3c). SIRT6 transcripts were reduced in Panc-1shKLF10 cells (Fig. 3d, right lower panel). The expression of SIRT6 paralleled that of KLF10 in genetically manipulated PDAC cell lines, including Panc-1, BXPC3, and MiaPaCa cells (Fig. 3d). In the PDAC tissue of 29 patients, the KLF10 and SIRT6 levels were reduced in parallel compared with the levels in the normal pancreas, with a correlation coefficient (*r*) = 0.54 (*p* = 0.003; Fig. 3e, f). There was a moderately positive correlation between KLF10 and SIRT6 from the Oncomine data (Fig. 3g).

### SIRT6 is an essential mediator of the regulatory effects of KLF10 on EMT, glycolysis, and the metastasis of pancreatic cancer

**Fig. 4:**
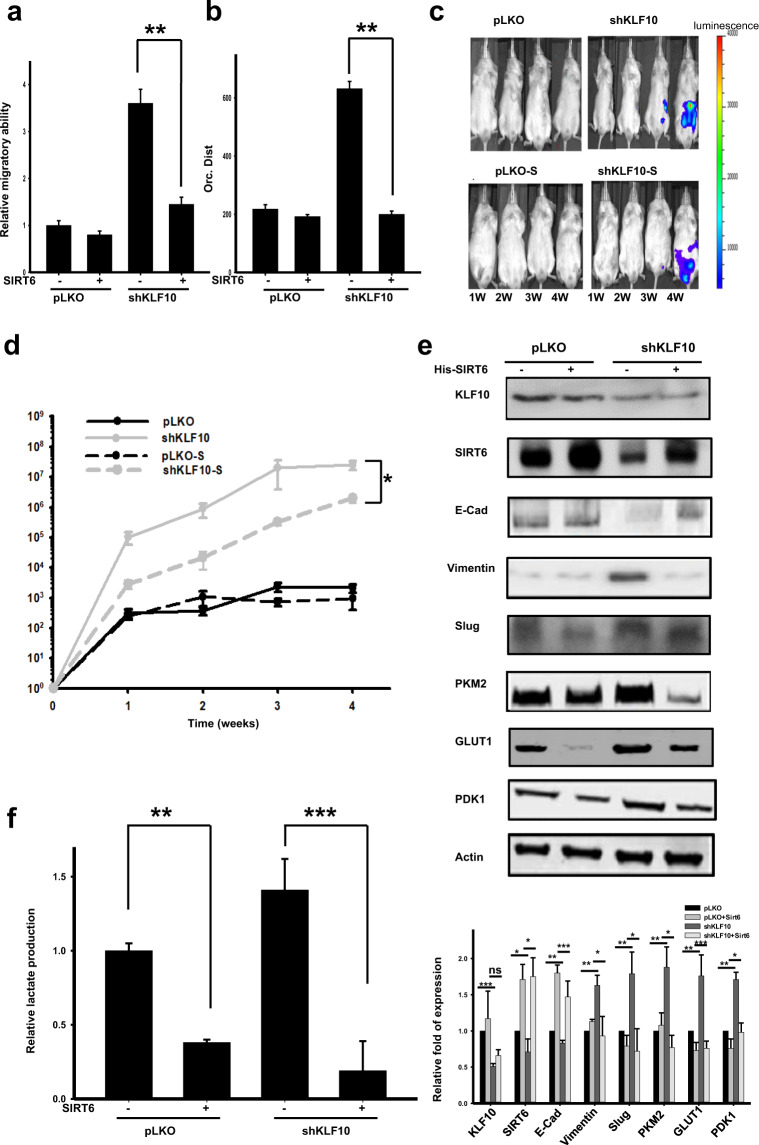
Overexpression of SIRT6 ameliorated the migration, EMT phenotype, glycolysis activity, and metastasis of PDAC. **a** Cumulated data of the Transwell migratory assay of Panc-1pLKO and Panc-1shKLF10 cells with and without SIRT6 overexpression. Data are presented as the mean ± SE (** signifies *p* < 0.01). The experiments were repeated three times. **b** Cumulated data of directional migration over time (or. dist.) in Panc-1pLKO and Panc-1shKLF10 cells with and without SIRT6 overexpression. Data are presented as the mean ± SE (** signifies *p* < 0.01). The cell trajectory experiments were repeated three times. **c** Representative IVIS images of mice at 1–4 weeks after injection with ASPC-1pLKO and ASPC-1shKLF10 cells with and without SIRT6 overexpression as indicated. **d** Cumulated IVIS signal of at least six mice in each experimental group injected with ASPC-1pLKO and ASPC-1shKLF10 with and without SIRT6 overexpression. Data are presented as the mean ± SE (* represents *p* < 0.05). **e** Representative immunoblots of E-cadherin and mesenchymal and glycolytic protein expression in Panc-1pLKO and Panc-1shKLF10 cells with and without SIRT6 overexpression. β-Actin was used as the internal control. Quantitative analysis of at least three experiments is shown below the immunoblots. **f** Cumulated lactate production assay data of Panc-1pLKO and Panc-1shKLF10 cells with and without SIRT6 overexpression. Data are presented as the mean ± SE (** and *** signify *p* < 0.01 and *p* < 0.005, respectively). The experiments were repeated three times.

Overexpressing SIRT6 in Panc-1shKLF10 cells reversed the migratory capacity induced by KLF10 deficiency (Fig. 4a, b; Supplementary Fig. 3a). Similar observations were noted in ASPC-1shKLF10 cells (Supplementary Fig. 3b). The murine metastatic model revealed the suppressed metastatic luminescent signal caused by SIRT6 overexpression in ASPC-1shKLF10 cells (Fig. 4c, d). The results were recapitulated in Panc-1 cells (Supplementary Fig. 3d, e). The mesenchymal protein level was reduced, and E-cadherin expression was increased by overexpressing SIRT6 in Panc-1shKLF10 cells (Fig. 4e). Glycolysis activity was reduced by the forced expression of SIRT6 in Panc-1shKLF10 or ASPC-1shKLF10 cells with reduced lactate production and downregulation of PDK1, PKM2, and Glut1 (Fig. 4e, f; Supplementary Fig. 3c). We concluded that SIRT6 was an essential downstream mediator of KLF10 and that it contributed to EMT and the glycolysis phenotypes of PDAC.

### Linoleic acid (LA) increased SIRT6 expression and reversed the malignant phenotype of PDAC

**Fig. 5:**
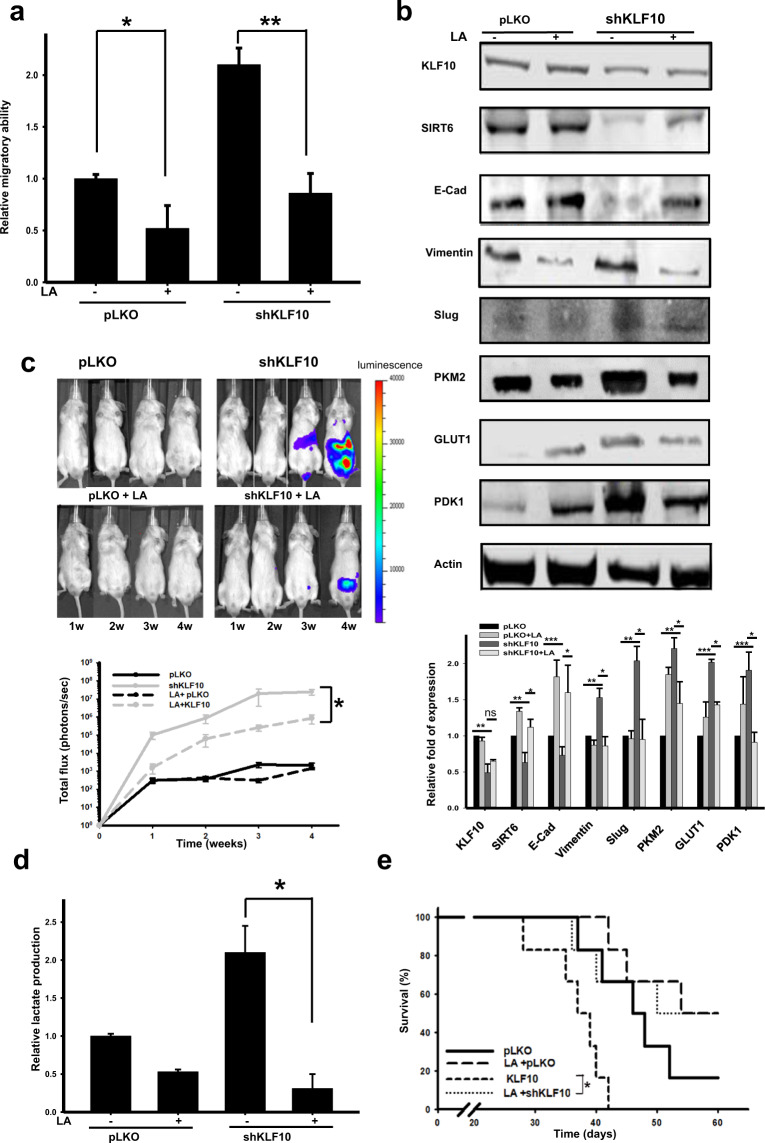
Linoleic acid (LA) enhanced SIRT6 expression and reversed migration, EMT, and the glycolytic phenotypes of PDAC with KLF10 deficiency. **a** Cumulative migratory assay of Panc-1pLKO and Panc-1shKLF10 cells with and without 50 μM LA treatment for 16 h. Data are presented as the mean ± SE (* and ** represent *p* < 0.05 and *p* < 0.01, respectively). The experiments were repeated three times. **b** Representative immunoblots of E-cadherin and mesenchymal and glycolytic protein expression in Panc-1pLKO and Panc-1shKLF10 cells with and without 50 μM LA treatment for 16 h. β-Actin was used as the internal control. Quantitative analysis of at least three experiments is shown below the immunoblots. **c** Upper panel: Representative IVIS image of mice at 1–4 weeks after injection with ASPC-1pLKO and ASPC-1shKLF10 with and without 1% LA treatment in daily water for 8 weeks as described in the “Materials and methods”. Lower panel: Cumulated IVIS signal of at least six mice in each experimental group that were injected with ASPC-1pLKO and ASPC-1shKLF10 and received 1% LA treatment in daily water for 8 weeks or did not, as indicated. Each point represents the mean ± SE at least six mice (* signifies *p* < 0.05). **d** Cumulated lactate production assay of Panc-1pLKO and Panc-1shKLF10 cells with and without LA treatment at 50 μM for 16 h. Data are presented as the mean ± SE (* represents *p* < 0.05). The experiments were re*p*eated three times. **e** The survival curves of mice injected with Panc-1pLKO and Panc-1shKLF10 that received 1% LA treatment in daily water for 8 weeks or did not, as indicated. Each experimental group contained at least six mice. The median survival of mice injected with Panc-1shKLF10 without and with LA treatment was 37 and 50 days (*p* = 0.038; * signifies *p* < 0.05).

LA, which is a polyunsaturated omega-6 fatty acid, is one of two essential fatty acids^21^. A recent investigation reported that LA at physiological concentrations induces a conformational change in SIRT6 but not in other sirtuins and increases catalytic efficiency^12^. We unexpectedly found that LA induced a dose-dependent increase in SIRT6 expression levels but not other sirtuin expression levels in PDAC cells (Supplementary Fig. 4a, b). The migratory ability and mesenchymal protein expression of Panc-1shKLF10 were suppressed by LA (Fig. 5a, b). The luminescent signals of the murine metastatic model with ASPC-1shKLF10 or Panc-1shKLF10 were suppressed by LA treatment (Fig. 5c; Supplementary Fig. 4c, d). LA treatment in Panc-1shKLF10 cells revealed downregulated lactate production and glycolytic enzyme expression (Fig. 5b, d). SIRT6 expression was elevated by LA in murine tumor tissues (Supplementary Fig. 4e). The survival of mice implanted with Panc-1shKLF10 was prolonged significantly with LA treatment in daily water (median survival time from 37 to 50 d, *p* = 0.038; Fig. 5e).

### Interaction of EMT and glycolysis in pancreatic cancer with KLF10 loss

**Fig. 6:**
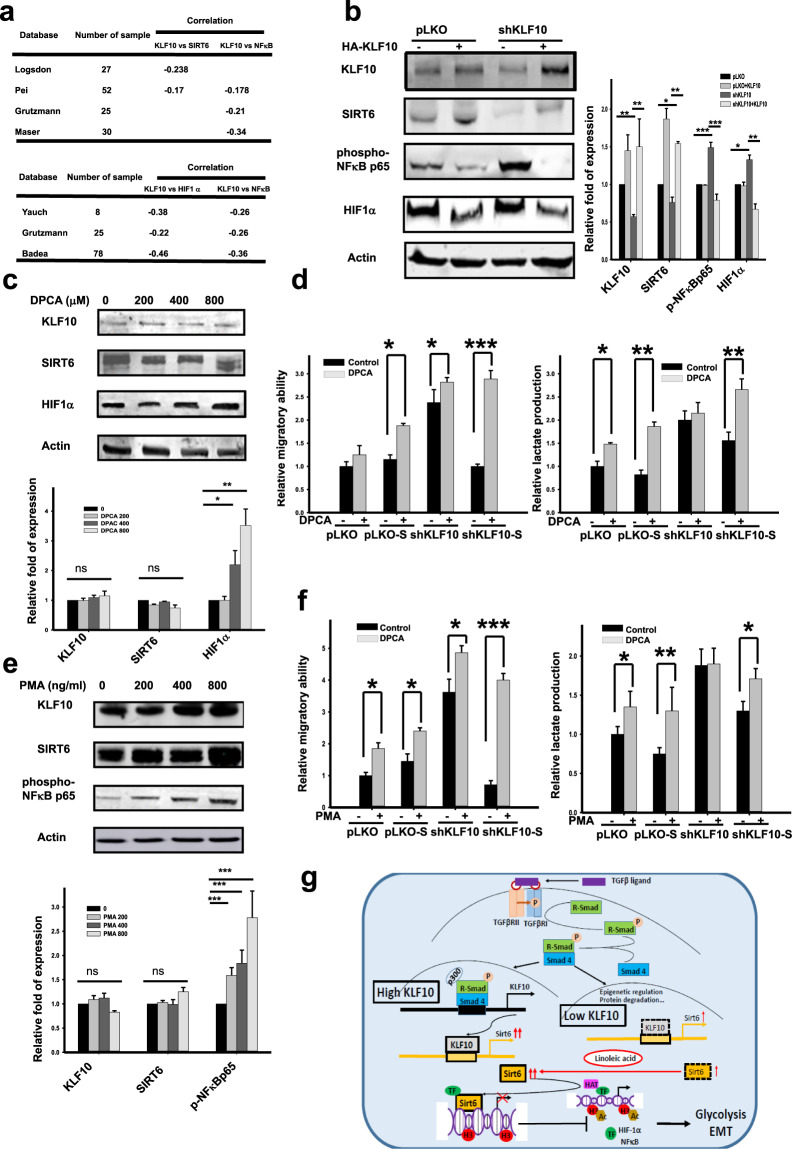
HIF1α and NFκB are two downstream mediators of KLF10/SIRT6 signaling in PDAC cells. **a** Representative databases from Oncomine display the correlation between KLF10 (upper panel)/SIRT6 (lower panel) and HIF1α or NFκB transcripts. **b** Representative immunoblots of HIF1α and phospho-NFκBp65 in Panc-1pLKO and Panc-1shKLF10 cells with and without forced expression of KLF10. Quantitative analysis of cumulative data from at least three experiments is shown in addition to immunoblots. β-Actin was used as the internal control. **c** Representative immunoblots of HIF1α in Panc-1 cells treated with various dosages of 1,4-dihydrophenonthrolin-4-one-3-carboxylic acid (DPCA) for 8 h. Quantitative analysis from at least two experiments is shown below the immunoblots. **d** Left panel: Cumulated migratory assay of Panc-1pLKO and Panc-1shKLF10 cells with or without SIRT6 overexpression treated with and without 400 μM DPCA for 16 h. Right panel: Cumulated lactate production assay of Panc-1pLKO and Panc-1shKLF10 cells with and without SIRT6 overexpression that were treated with and without 400 μM DPCA for 16 h. Data are presented as the mean ± SE (*n* = 3) (*, ** and *** indicate *p* < 0.05, *p* < 0.01, and *p* < 0.005, respectively). The experiments were repeated three times. **e** Representative immunoblots of NFκΒ in Panc-1 cells treated with various dosages of phorbol ester (PMA) for 8 h. Quantitative analysis from at least two experiments is shown below the immunoblots. **f** Left panel: Cumulated migratory assay of cells treated with and without 200 ng/ml PMA for 16 h. Right panel: Cumulated lactate production assay with and without 400 ng/ml PMA for 16 h. Data are presented as the mean ± SE (*n* = 3; *, **, and *** indicate *p* < 0.05, *p* < 0.01 and *p* < 0.005, respectively). The ex*p*eriments were repeated three times. **g** Graphic summary of the role of the KLF10/SIRT6 signaling pathway in modulating EMT and glycolysis in PDAC.

HIF1α and NFκΒ were two candidate mediators downstream of the KLF10/SIRT6 signal^22,23^. From the Oncomine database, we noted a moderate inverse correlation between KLF10/SIRT6 and HIF1α and NFκB transcripts (Fig. 6a). Moderate correlations of HIF1α or NFκB with glycolytic enzymes or EMT molecules, respectively, were also noted (Supplementary Fig. 5a, b). In our study, the expression levels of NFκΒ and, to a lesser extent, HIF1α, were upregulated in Panc-1shKLF10 cells with low levels of SIRT6 (Fig. 6b). Forced expression of KLF10 reduced the expression of HIF1α and NFkΒ and upregulated SIRT6 (Fig. 6b). Using DPCA^24^ and PMA to activate HIF1α and NFκB, respectively (Fig. 6c, e), the ameliorating effects on migration and lactate production by overexpressing SIRT6 in Panc-1shKLF10 cells were abolished (Fig. 6d, f).

In summary, (Fig. 6g), KLF10 coordinated EMT and glycolysis activity in PDAC through the use of SIRT6. In our model system, NFκΒ and possibly HIF1α were responsible for the interaction between glycolysis activity and EMT phenotype downstream of the KLF10/SIRT6 signal.

## Discussion

Distant metastasis is the major cause of death in PDAC, which is known for its aberrant TGFβ signaling. However, the role of TGFβ in cancer proliferation and metastasis is complex and elusive. KLF10, a TGFβ signal early responder, has antiproliferative and antimetastatic effects in contrast to TGFβ and is a potential target for developing therapeutic targets for PDAC. Our previous study revealed that elevating KLF10 genetically or pharmacologically by metformin may reverse the radioresistance of PDAC^25^. In this study, we discovered that KLF10 transcriptionally activated SIRT6 to coordinate EMT and glucose metabolism in PDAC. Genetic overexpression of SIRT6 or pharmacological use of LA reversed the EMT and glycolysis phenotypes and suppressed the distant metastasis of PDAC. In contrast to targeting TGFβ, the therapeutic strategy of upregulating KLF10/SIRT6 signaling in PDAC may reduce distant metastasis without enhancing tumor proliferation.

The KLF family consists of 18 identified members with diverse regulatory roles in glycolysis, EMT, and metastasis. Redundant subgroups within the KLF family are emerging^26,27^. Despite a significant role of KLF10 in modulating EMT and glycolysis in PDAC^7,8^, the interplay among various KLF transcripts that balances opposing outcomes of epithelial homeostasis and glucose metabolism remains unclear. A previous study identified KLF10 as a rheostat that controls TGFβ signaling and EMT activation by transcriptionally regulating Slug^7^. In our study, SIRT6 overexpression in KLF10-deficient PDAC cells reduced Slug and other EMT transcription proteins. The results indicated that in addition to directly modulating Slug transcriptionally, KLF10 coordinated EMT transcription, including Slug, and glucose homeostasis using SIRT6.

In contrast to most studies, KLF10, a transcriptional repressor, was found to transcriptionally activate SIRT6 in pancreatic cancer cells. Sp1-like/KLF transcription factors function as activators or repressors depending on which promoter they bind and the coregulators with which they interact^28^. Class III Sp1-like/KLF family members, including KLF10, share a conserved repression motif that is sufficient to mediate transcriptional repression by interacting with the histone deacetylase corepressor complex mSin3A. However, this function can be modified by cell signaling events. Phosphorylation of four residues in a region adjacent to the SID-like domain involving extracellular regulator kinase 2 disrupts interaction with mSin3A and results in a significant loss of repressor function^29^. Recent evidence also suggests that histone acetylation and deacetylation may serve as a switch for Sp1-like/KLF proteins to function as activators or repressors^28^. The molecular mechanism by which KLF10 switches between transcriptional activation and suppression warrants further study.

SIRT6 belongs to the sirtuin family of NAD^+^-dependent deacetylases involved in stress resistance and metabolic homeostasis^11^. Recent studies have reported that SIRT6 functions as a key regulator of glucose homeostasis and as a tumor suppressor^30^. SIRT6 has been reported to inhibit Twist 1 and EMT in lung cancer and idiopathic pulmonary fibrosis, respectively^31,32^. On the other hand, studies have also shown SIRT6 to enhance cancer metastasis by promoting EMT in lung, thyroid, and liver cancer^33–35^. The controversial role of SIRT6 in the pathogenesis of cancers may be tissue- and context-dependent. Tian K et al.^32^ demonstrated that SIRT6 activates TGFβ1/Smad3 signaling and suppresses EMT in bleomycin-injured mice. In our PDAC model, KLF10, a TGFβ downstream mediator, transcriptionally activated SIRT6 and inhibited EMT transcription factors, probably by using NFκB and HIF1α. Overexpressing SIRT6 genetically or pharmacologically did not significantly change the KLF10 expression level in our model system. Whether feedback signals occur between KLF10 and SIRT6 merits exploration. A growing body of evidence identifies sirtuins as key epigenetic modulators of EMT activation and maintenance^36^. Given the opposing roles of sirtuins in cancer, future investigations that focus on the development of selective biomarkers and precision therapies for individual cancer types are crucial.

Metabolic reprogramming is a fundamental driver of initiating EMT in cancer^37^. Recently, an alternative concept has emerged that suggests that the primary function of activated oncogenes and inactivated tumor suppressors is to reprogram nutrient uptake and cellular metabolism^38^. By using DPCA, a HIF1α activator, and PMA, an NFκB activator, we activated glycolysis and EMT phenotypes in PDACshKLF10 cells that overexpressed SIRT6. The findings indicated that NFκB and HIF1α are potential downstream mediators of KLF10/SIRT6 governing both glucose homeostasis and EMT in PDAC.

Given that SIRT6 is a poor deacetylase and preferentially binds to long-chain acylated peptides to hydrolyze them, certain free fatty acids, including myristic, oleic, and LA, may stimulate deacetylation activity^12^. In this study, LA was used at physiological concentrations to induce a conformational change that activated SIRT6 acetylation activity, which was not observed with other sirtuins^12^. A recent study revealed that nitrated fatty acids with a Michael adduct resulted in 20-fold stronger activation of SIRT6^39^. An unexpected finding was that the SIRT6 protein level was elevated by LA treatment in our model. A possible explanation is that free fatty acids may inhibit cellular proteasome activity and stabilize LA protein levels^40,41^. In our vector control pancreatic cancer cells, the levels of glycolytic enzymes were significantly elevated by LA but were suppressed by genetically overexpressing SIRT6. A possible explanation is that SIRT6 overexpression is selectively toxic to multiple cancer cells, and there are multiple ways to fine-tune SIRT6 levels in cancer cells in response to stress conditions^11,42^. In KLF10-deficient pancreatic cancer cells with low SIRT6 levels, LA elevated SIRT6 expression and activity, which led to upregulated glycolytic enzymes. However, in wild-type pancreatic cancer cells with sufficient amounts of SIRT6, the effect of LA in elevating SIRT6 was less efficient. On the other hand, LA may induce PGC-1α to increase glucose transport and lipolysis^43^, which leads to elevated glycolytic enzymes.

Replacing saturated fat with polyunsaturated fat reduced blood cholesterol concentrations and the risk of coronary artery disease. However, some animal studies have indicated that LA may promote tumor growth in murine models. Current evidence, including meta-analysis, does not support the increased risk of cancer by LA^44,45^. In our in vitro study, the inconsistent effect of overexpressing SIRT6 and LA on glycolytic enzyme levels in Panc-1pLKO cells (Figs. 4e and 5b) suggests a complicated effect of LA on glycolytic activity in PDAC cells without KLF10 deficiency. Benefits from the long-term consumption of LA in preventing PDAC progression warrant further investigation.

In our study, significant upregulation of NFκB and, to a lesser extent, HIF1α expression, was noted by interfering with the KLF10/SIRT6 signal. A previous study revealed that SIRT6 deacetylates histone H3 lysine 9 on promoters of NFκB target genes to destabilize NFκB^46^. SIRT6 was reported to be a corepressor of the transcription factor HIF1α. SIRT6-deficient cells exhibited increased HIF1α activity and protein stability with upregulation of glycolysis^23^. Our in vitro studies using pharmacological manipulation of HIF1α and NFκB revealed an interaction between the EMT and glycolysis phenotypes of PDAC cells without affecting the KLF10/SIRT6 expression levels. The results suggested that NFκB and HIF1α were two candidate downstream mediators of KLF10/SIRT6 signaling that regulate EMT and glycolysis in PDAC (Fig. 6e).

Distinguishing between the antiproliferative and prometastatic functions of TGFβ makes KLF10 a potential target for the development of therapies for PDAC. We demonstrated the antimetastatic effect of KLF10 and discovered a novel pathway by which KLF10 coordinates EMT and glycolysis by transcriptionally regulating SIRT6 in PDAC. KLF10/SIRT6 signaling modulates EMT proteins and glycolysis enzymes using NFκB and probably HIF1α. Upregulating SIRT6 genetically or pharmacologically using LA may mitigate EMT, glycolysis, and distant metastasis of PDAC due to KLF10 deficiency.

## Supplementary information

Supplementary Information

## Data Availability

The datasets supporting the conclusions of this article are included within the article and its Supplementary files.
