# Supplementary material for: Upregulating sirtuin 6 ameliorates glycolysis, EMT and distant metastasis of pancreatic adenocarcinoma with krüppel-like factor 10 deficiency
Source: Exp Mol Med. 2021 Oct 26;53(10):1623–35. doi: 10.1038/s12276-021-00687-8 (PMC8569177; doi:10.1038/s12276-021-00687-8)
Supplement: Supplementary file 1 — Supplementary Information [file 12276_2021_687_MOESM1_ESM.pdf]

Supplementary Information

Upregulating sirtuin 6 ameliorates glycolysis, EMT and distant metastasis of pancreatic adenocarcinoma with krüppel-like factor 10 deficiency

Yi-Chih Tsai[1], Su-Liang Chen[1], Shu-Ling Peng[2], Ya-Li Tsai[1], Zuong-Ming Chang[1], Vincent Hung-Shu Chang[3], Hui-Ju Ch'ang[1,4,5]

[1]National Institute of Cancer Research, National Health Research Institutes, Zhunan, Taiwan; [2]Department of Pathology, National Cheng Kung University Hospital, Tainan, Taiwan; [3]Program for Translation Biology, [4]Program for Cancer Biology and Drug Discovery, College of Medical Science and Technology, Taipei Medical University, Taiwan; [5]Department of Oncology, School of Medicine, College of Medicine, National Cheng Kung University, Tainan, Taiwan

Corresponding author: Hui-Ju Ch'ang M.D.
ORCID iD: https://orcid.org/0000-0002-3706-1769
R1-2034, 35 Keyan road, Zhunan, Miaoli County 35053, Taiwan
Tel: 886-37-246-166 ext. 35105; Fax: 886-37-586-463; E-mail: hjmc@nhri.org.tw

Supplementary figures

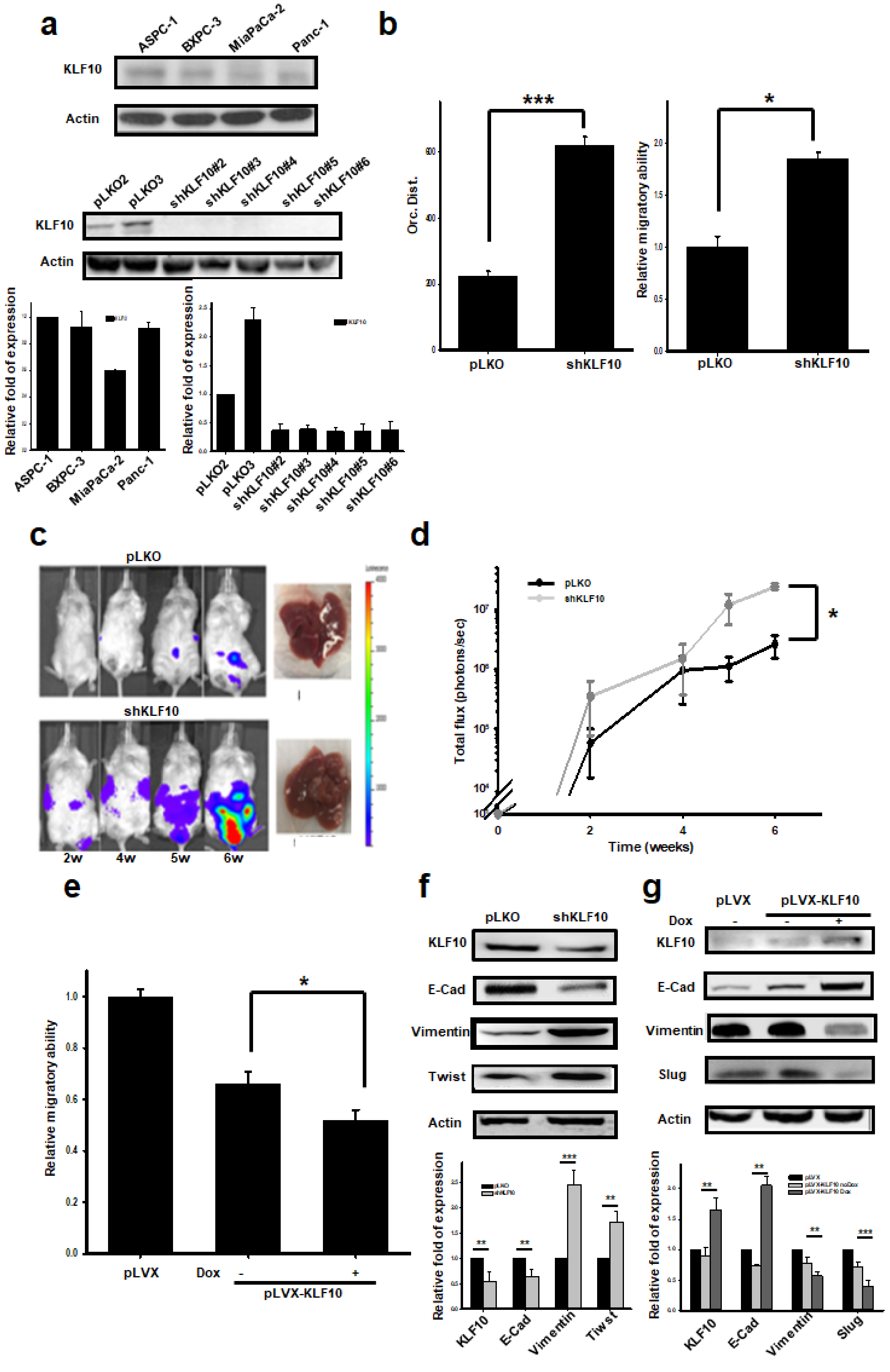

Supplementary Figure 1: (a) Upper panel: Representative immunoblots of KLF10 expression in pancreatic cancer cell lines. Lower panel: Representative immunoblots of five stable clones of Panc-1shKLF10 (shKLF10 #2-6) generated by the most efficient KLF10 targeting shRNA constructs as mentioned in Materials and Methods. pLKO2 and 3 are two vector control Panc-1 cells. Quantitative analysis cumulated from at least two experiments was shown below immunoblots. (b) Left panel: Cumulated data of net displacement from origin (or. dist.) of Panc-1pLKO and Panc-1shKLF10 described in Figure 1c, upper panel (∗∗∗ signifies $p < 0.005$). The experiments were repeated independently three times. Right panel: Cumulated migratory assay of ASPC-1pLKO and ASPC-1shKLF10. Data are presented as mean ± SE (∗ signifies $p < 0.05$). The experiments were repeated three times. (c) Representative IVIS images of mice on 2, 4, 5, 6 weeks after injection with Panc-1pLKO or Panc-1shKLF10. Representative liver specimens with tumor nodules are shown on the right panel. (d) Cumulated IVIS signal of at least six mice in each group injected with Panc-1pLKO or Panc-1shKLF10 over time. Data were presented as mean ± SE. (∗signifies $p < 0.05$) (e) Cumulated migratory assay of MiaPaCa cells with or without KLF10 overexpression induced by doxycycline (Dox). Data are presented as mean ± SE (∗ signifies $p < 0.05$). The experiments were repeated three times. (f) Representative immunoblots of mesenchymal proteins and E-cadherin on ASPC-1pLKO and ASPC-1shKLF10. Quantitative analysis cumulated from at least two experiments was shown below immunoblots. β-actin was used as the internal control. (g) Representative immunoblots of E-cadherin and mesenchymal proteins expression on MiaPaCa cells with and without KLF10 overexpression induced by Dox. Quantitative analysis cumulated from at least two experiments was shown below immunoblots. β-actin was used as the internal control.

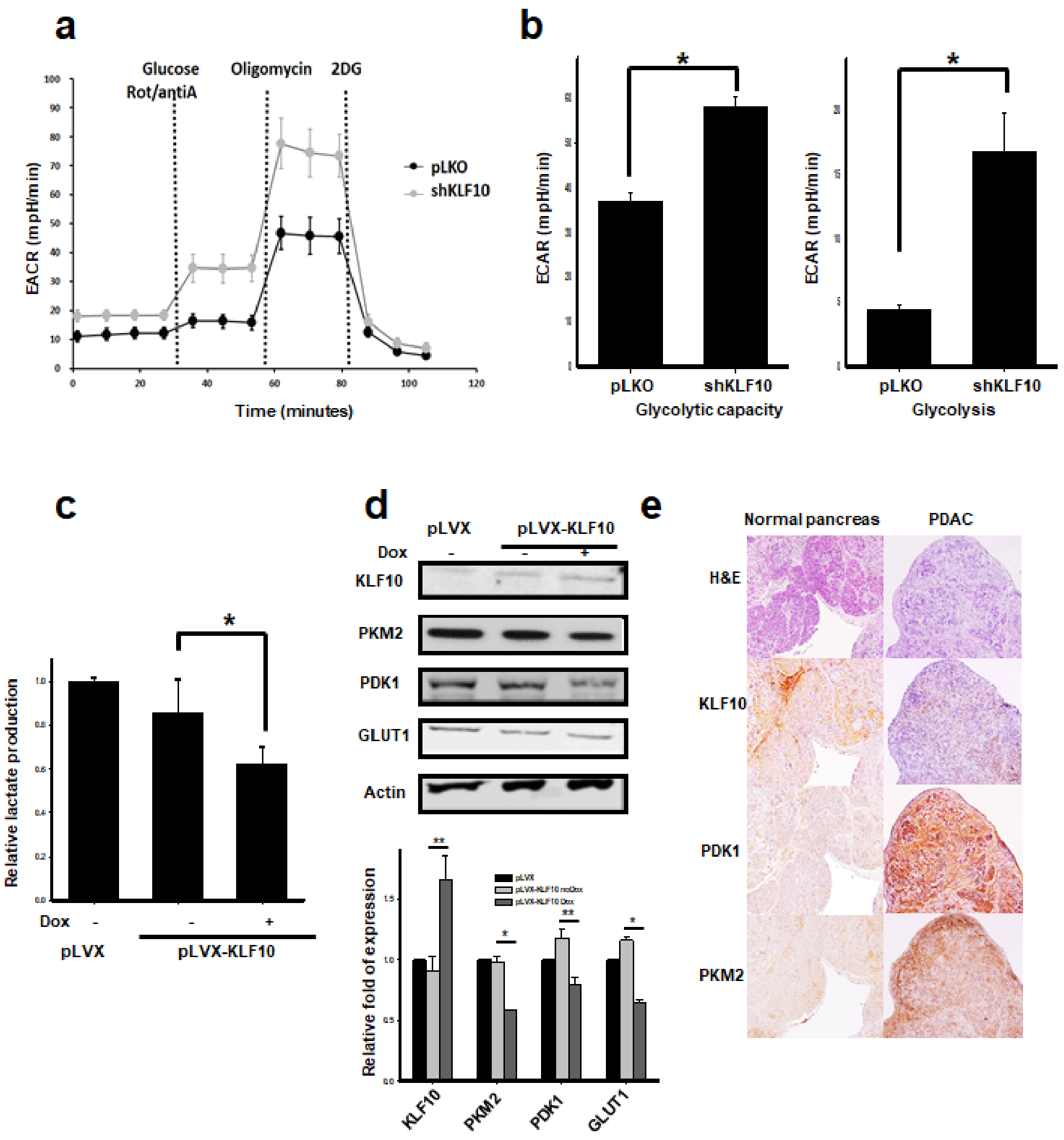

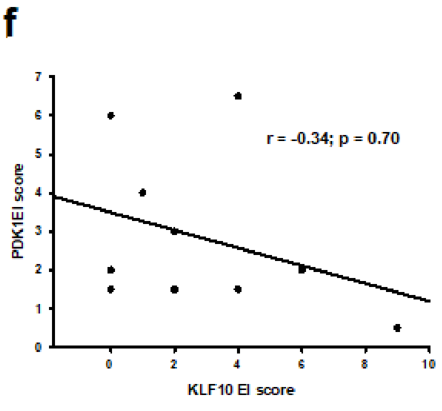

g

Database	Number of sample	Correlation of KLF10 vs PDK1
Lonsdon	27	-0.119
Buchholz	38	-0.28
Segara	17	-0.21
Pei	52	-0.28

Supplementary Figure 2: (a) Extracellular acidification rate (ECAR) in Panc-1pLKO vs Panc-1shKLF10 in response to successive injection of glucose, oligomycin and 2-DG. Results were normalized according to cell number and presented as mean ± SE ($n = 3$). (b) Basal (right panel) and maximal (left panel) glycolytic capacities were determined in the same experimental setting for Panc-1pLKO vs Panc-1shKLF10. Data are presented as mean ± SE ($n = 3$; ∗ signifies $p < 0.05$). (c) Cumulated lactate production assay of MiaPaCa cells with and without KLF10 overexpression induced by Dox. Data are presented as mean ± SE (∗ signifies $p < 0.05$). The experiments were repeated three times. (d) Representative immunoblots of glycolytic enzymes on MiaPaCa cells with and without KLF10 overexpression induced by Dox. Quantitative analysis cumulated from at least two experiments was shown below immunoblots. β-actin was used as the internal control. (e) Representative H&E, immunohistochemistry of KLF10, PKM2, PDK1 on non-tumor part normal pancreas and pancreatic tumor tissue from KC mice of 18 week-old. (f) Correlation of KLF10 and PDK1 expression in pancreatic tissue from ten patients, from the cohort mentioned in Materials and Methods, with curatively resected PDAC. The correlation coefficient = -0.34, $p = 0.70$. (g) Representative four databases from Oncomine showing negative correlation of Klf10 and PDK1.

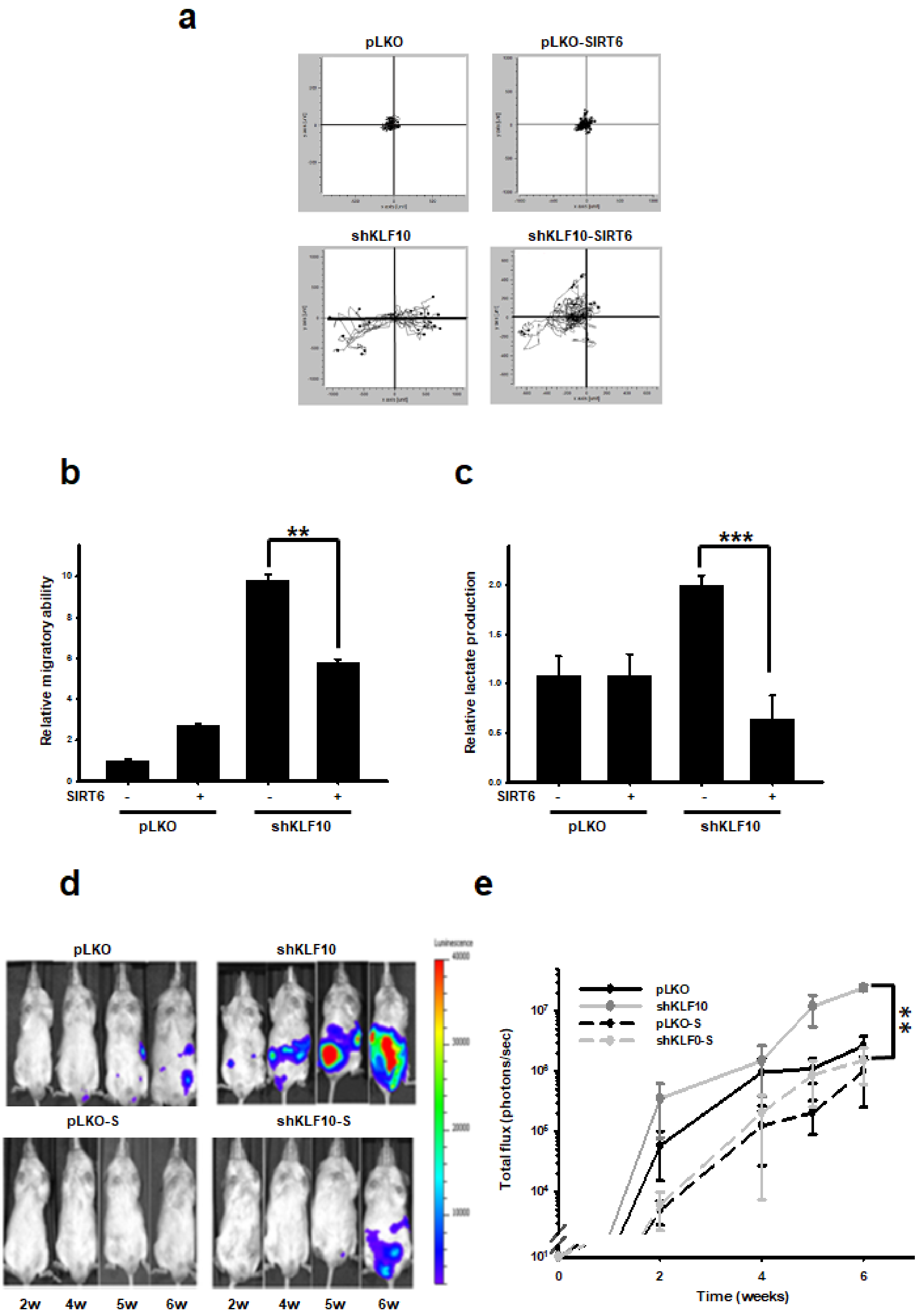
a
pLKO
pLKO-SIRT6
shKLF10
shKLF10-SIRT6
b
**
Relative migratory ability
SIRT6 - + - +
pLKO
shKLF10
c
***
Relative lactate production
SIRT6 - + - +
pLKO
shKLF10
d
pLKO
shKLF10
pLKO-S
shKLF10-S
2w 4w 5w 6w 2w 4w 5w 6w
e
pLKO
shKLF10
pLKO-S
shKLF0-S
Total flux (photons/sec)
Time (weeks)
**

Supplementary Figure 3: (a) Cumulated plots of cell trajectory data of twenty cells from Panc-1pLKO and Panc-1shKLF10 with and without SIRT6 overexpression as indicated. (b) Cumulated migratory assay of ASPC-1pLKO and ASPC-1shKLF10 with and without SIRT6 overexpression. Data are presented with mean ± SE (∗∗ signifies $p < 0.01$). The experiments were repeated three times. (c) Cumulated lactate production assay of ASPC-1pLKO and ASPC-1shKlf10 with or without SIRT6 overexpression. Data are presented with mean ± SE (∗∗∗ signifies $p < 0.005$). The experiments were repeated three times. (d) Representative IVIS image of mice on 2, 4, 5, 6 weeks after injection with Panc-1pLKO and Panc-1shKLF10 with and without SIRT6 overexpression. (e) Cumulated data of IVIS signal of at least six mice in each group after injection with Panc-1pLKO or Panc-1shKLF10 with and without SIRT6 overexpression over time. Data are presented with mean ± SE (∗∗ signifies $p < 0.01$).

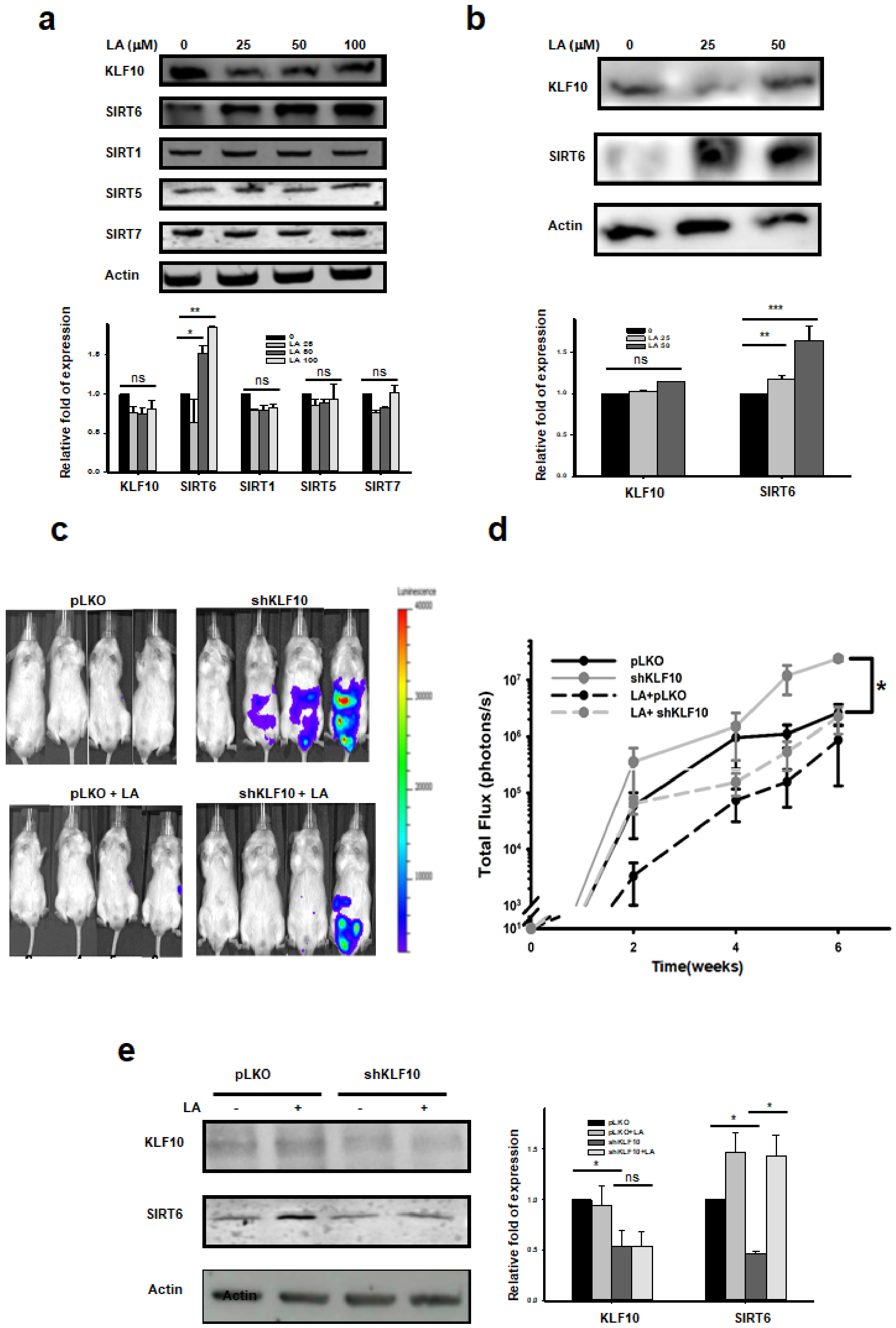

Supplementary Figure 4: (a) Representative immunoblots of KLF10 and sirtuins in Panc-1 cells after various dosages of LA for 16h. Quantitative analysis cumulated

from at least two experiments was shown below immunoblots. β-actin was used as the internal control. (b) Representative immunoblots of SIRT6 on ASPC-1 cells treated with various dosages of LA for 16h. Quantitative analysis cumulated from at least two experiments was shown below immunoblots. β-actin was used as the internal control. (c) Representative IVIS images of mice on 2, 4, 5, 6 weeks after injection with Panc-1pLKO or Panc-1shKLF10 with and without 1.5% LA treatment in daily water for 8 weeks as described in Materials and Methods. (d) Cumulated IVIS signal of at least six mice in each group injected with Panc-1pLKO or Panc-1shKLF10 with or without 1.5% LA treatment in daily water for 8 weeks. Data are presented with mean ± SE (∗ signifies $p < 0.05$). (e) Representative immunoblots of KLF10 and SIRT6 expression of tumor lysates from mice, implanted with Panc-1pLKO and Panc-1shKlf10, receiving 1% LA treatment or not as described in Materials and Methods. Quantitative analysis cumulated from two experiments was shown beside immunoblots. β-actin was used as the internal control.

a

Database	Number of sample	Correlation of HIF1α vs glycolysis enzymes		
		PKM2	PDK1	GLUT1
Badea	78	0.82	0.27	0.43
Collisson	20	0.12	0.33	0.4
Iacobuzio-Donahue	36		0.45	0.53
Ishikawa	49		0.3	0.29

Database	Number of sample	Correlation of HIF1α vs EMT molecules		
		E-cadherin	Vimentin	Slug
Badea	78	-0.26	0.78	0.84
Grutzmann	25	-0.64	0.48	0.41
Iacobuzio-Donahue	36	-0.23	0.35	0.34

b

Database	Number of sample	Correlation of NFκB vs glycolysis enzymes		
		PKM2	PDK1	GLUT1
Badea	78	0.4	0.28	0.62
Iacobuzio-Donahue	36		0.2	0.46

Database	Number of sample	Correlation of NFκB vs EMT molecules		
		E-cadherin	Vimentin	Slug
Badea	78	-0.47	0.7	0.884
Iacobuzio-Donahue	36		0.2	0.53
Maser	30	-0.52	0.3	

Supplementary Figure 5: (a) Databases from Oncomine correlating levels of HIF1α with glycolytic enzymes (upper panel) or EMT molecules (lower panel). (b) Databases from Oncomine correlating levels of NFκB with glycolytic enzymes (upper panel) or EMT molecules (lower panel).
